# Mechanisms of long noncoding RNA function in development and disease

**DOI:** 10.1007/s00018-016-2174-5

**Published:** 2016-03-23

**Authors:** Sandra U. Schmitz, Phillip Grote, Bernhard G. Herrmann

**Affiliations:** Department of Developmental Genetics, Max Planck Institute for Molecular Genetics, Ihnestr. 63-73, 14195 Berlin, Germany; Institute of Cardiovascular Regeneration, Center for Molecular Medicine, Goethe University, Theodor-Stern-Kai 7, 60590 Frankfurt, Germany; Institute for Medical Genetics, Campus Benjamin Franklin, Charite-University Medicine Berlin, Hindenburgdamm 30, 12203 Berlin, Germany

**Keywords:** LncRNA, Differentiation, Cardiovascular disease, Cancer, Gene regulation, Chromatin, Epigenetics, Genome organization

## Abstract

Since decades it has been known that non-protein-coding RNAs have important cellular functions. Deep sequencing recently facilitated the discovery of thousands of novel transcripts, now classified as long noncoding RNAs (lncRNAs), in many vertebrate and invertebrate species. LncRNAs are involved in a wide range of cellular mechanisms, from almost all aspects of gene expression to protein translation and stability. Recent findings implicate lncRNAs as key players of cellular differentiation, cell lineage choice, organogenesis and tissue homeostasis. Moreover, lncRNAs are involved in pathological conditions such as cancer and cardiovascular disease, and therefore provide novel biomarkers and pharmaceutical targets. Here we discuss examples illustrating the versatility of lncRNAs in gene control, development and differentiation, as well as in human disease.

## Introduction

It has long been known that several classes of non-protein-coding RNA molecules exert important cellular functions. For instance, ribosomal RNAs (rRNAs) are essential elements of the translation machinery and small nuclear RNAs (snRNAs) are required for splicing of nascent RNA transcripts. Also, various classes of small (around 20–30 nucleotides) noncoding RNAs such as micro (mi)RNAs, small inhibitory (si)RNAs or PIWI interacting (pi)RNAs are well known as gene silencers. With the recent advent of massive parallel sequencing techniques, however, it has been observed that a tremendously high portion, approximately 70 %, of the genome is transcribed in various contexts and cell types [1, 2]. A large proportion of these newly detected RNA transcripts are structurally indistinguishable from protein-coding and processed messenger RNAs (mRNAs). They tend to be expressed at a very low level and have little to no protein-coding potential. This subclass of noncoding transcripts of variable length and function is collectively referred to as long noncoding RNAs (lncRNAs).

A plethora of biological tissues, organs, pathological samples and cultured cells have been analyzed for noncoding RNA expression, and it is clear that these molecules are omnipresent. Apparently, defining noncoding RNA function has proven more challenging than detecting them, as the number of reports showing comprehensive functional data is far smaller than those describing their identification in various contexts. The flexibility of RNA transcripts and their ability to fold into complex 3D-conformations enables them to form specific interactions with proteins. They can interact with RNA or DNA molecules via base pairing, even with double-stranded DNA, and form networks with DNA, protein complexes and RNA molecules, illustrating their large potential as an important player with many biological functions. In this review, we will discuss mechanisms of lncRNA functions with a focus on their role in development and disease (Table 1).

**Table 1:** List of lncRNAs and their main features mentioned in this review

Name	Genomic category	Neigh-boring gene	Cellular localisation	Mechanism	Physiological/pathological setting	References
*AIRN*	Antisense/overlapping	*IGF2R*	Nucleus	Transcription	Imprinting	[118]
*ANCR (DANCR)*	LincRNA, miRNA host gene	*ERVMER34*	Nucleus	Histone modification	Epidermal differentiation	[139, 140]
*ANRIL*	Antisense	*CDKN2B*	Nucleus	Histone modification	Different cancer types, CVD	[58, 59, 169]
*BCAR4*	Divergent lncRNA	*RSL1D1*	Nucleus	Histone modification	Breast cancer	[82]
*BRAFP1* (*Braf*-*rs1*)	Pseudo gene	*ZDHHC15*	Cytoplasm	Post transcriptional	DLBCL	[158]
*Braveheart (Bvht)*	LincRNA	*IL17b*	Nucleus	Histone modification	Cardiac differentiation	[45]
*CDR1*	CircRNA	*CDR1*	Cytoplasm	Post transcriptional	Neuronal tissue	[26, 27]
*DACOR1* (*TCONS_00023265*)	LincRNA	*SMAD3*	Nucleus	DNA methylation	Colon cancer	[74]
*DEANR1*, *ALIEN*	LincRNA	*FOXA2*	Both	Transcription factor	Endoderm and cardiac differentiation	[76, 77]
*ecCEBPA*	Upstream lncRNA	*CEBPa*	Nucleus	DNA methylation	n.d.	[64]
*Evf2* (*Dlx6os*)	Divergent lncRNA/overlapping	*Dlx5/6*	Nucleus	Chromatin remodeling	Neuronal development	[69, 70]
*FAL1*	LincRNA (2 kb)	*ECM1*	Nucleus	Post transcriptional, histone modification	Ovarian cancer	[57]
*Fendrr*	Divergent lncRNA	*Foxf1*	Nucleus	Histone modification	Development	[47, 123]
*FIRRE*	LincRNA	*RNA5SP514*	Nucleus	3D genome organization	3D genome structure	[103]
*Gtl2* (*MEG3*)	LincRNA	*RTL1*	Nucleus	Histone modification, DNA methylation	Imprinting	[55, 122, 177]
*H19*	LincRNA	*IGF2/NCTC1*	Nucleus	DNA methylation	Imprinting, muscle differentiation	[116, 117]
*HOTAIR*	LincRNA	*HOXC11/HOXC12*	Nucleus	Histone modification	Different cancer types, skeletal development	[42–44, 152]
*HOTAIRM1*	Divergent lncRNA	*HOXA1*, *HOXA2*	n.d.	n.d.	Myeloid cancer cell lines	[178]
*Hotdog*	Enhancer RNA	*HoxD*	Nucleus	Enhancer	Development	[179]
*HOTTIP*	Divergent lncRNA	*HOXA13*	Nucleus	Histone modification	Limb development	[62]
*Jpx*	LincRNA	*Xist*	Nucleus	Transcription factor	X-chromosome inactivation	[83, 84]
*Lethe*	Pseudo gene	*Gmeb1*	Nucleus	Transcription factor	Inflammation	[79]
*Linc*-*HOXA1* (*Linc1547*, *HAUNT*)	LincRNA	*HOXA1*	Nucleus	Enhancer, histone modification	Development	[99, 123]
*Linc*-*P21*	LincRNA	*p21*	Nucleus	Transcription factor	Cancer, CVD	[123, 163, 165, 166]
*Lnc*-*DC*	LincRNA	*HEATR6*	Cytoplasm	Transcription factor	Dendritic cell differentiation	[81]
*LncMyoD*	LincRNA	*Munc*, *MyoD1*	Both	Post transcriptional	Muscle differentiation	[180]
*lncTCF7* (*WSPAR*)	LincRNA	*TCF7*	Nucleus	Chromatin remodeling	Hepatocellular carcinoma	[72]
*LUNAR1*	Divergent lncRNA	*PGPEP1L*,* IGF1R*	Nucleus	Transcription factor	Acute leukemia	[151]
*MALAT1 (NEAT2)*	LincRNA	*SCVL1*	Nucleus	Post transcriptional	Metastasis	[109, 110, 149]
*MiAT* (*Gomafu*, *RNCR2*)	LincRNA	*CRYBA4*	Nucleus	Post transcriptional	Myocardial infarction, neuronal differentiation, brain development, schizophrenia	[107, 108, 138, 167]
*MIR31HG*	LincRNA, miRNA host gene	*INK4A*	Nucleus	Histone modification	Senesence	[161]
*Myheart* (*Mhrt*)	Divergent lncRNA/overlapping	*Myh6/7*	Nucleus	Chromatin remodeling	Myocardial infarction	[68]
*NBAT*-*1*	Divergent lncRNA	*CASC15*	Nucleus	Histone modification	Neuronal differentiation, different tumors	[148, 150]
*NEAT1*	LincRNA	*FRMD8*	Nucleus	n.d.	Progesterone production/corpus luteum formation	[124, 127]
*NeST* (*Tmevpg1*)	LincRNA	*IFNg*	Nucleus	Histone modification	Infections	[63]
*NKILA*	Overlapping	*PMEPA1*	Cytoplasm	Transcription factor	Breast cancer	[153, 154]
*NORAD* (*LINC00657*)	LincRNA	*CNBD2*	Cytoplasm	Post transcriptional	Genomic stability	[111]
*PACER*	Divergent lncRNA	*COX2/PTGS2*	Nucleus	Transcription factor	Infection	[80]
*pancIL17d*	Divergent lncRNA	*IL17b*	Nucleus	DNA methylation	Preimplantation development	[66]
*PARTICLE*	Divergent lncRNA	*MAT2A*	Both	Histone modification	Increased in plasma from patients post-radiation	[54]
*PCGEM1*	LincRNA	*TMEF2*	Nucleus	Transcription factor, histone modification	Prostate cancer	[101, 144, 181]
*Pint*	Divergent lncRNA	*2210408F21Rik*	Nucleus	Histone modification	Colorectal cancer, growth/size	[48, 123]
*Pnky*	Divergent lncRNA	*Pou3f2*	Nucleus	Post transcriptional	Neuronal differentiation	[105]
*PRNCR1*	LincRNA	*CASC19*	Nucleus	Transcription factor, histone modification	Prostate cancer	[101, 181]
*RMST*	LincRNA	*NEDD1*	Nucleus	Transcription factor	Neuronal differentiation	[78, 129, 133]
*RNCR4*	Divergent lncRNA	*Mirc35HG*	Both	Post transcriptional	Retina development	[136]
*SChLAP1*	LincRNA	*UBE2E3*	Nucleus	Chromatin remodeling	Prostate cancer	[67]
*TARID*	Divergent lncRNA	*TCF21*	Nucleus	DNA methylation	Different cancer types	[65]
*TINCR*	LincRNA	*SAFb2*	Cytoplasm	Post transcriptional	Epidermal differentiation	[141, 142]
*Tsix*	LincRNA/antisense	*Xist*	Nucleus	Transcription factor, histone modification, chromatin remodeling	X-chromosome inactivation	[83]
*TUNA* (*megamind*, *Linc86023*)	LincRNA	*Tcl1*	Both	Post transcriptional	Pluripotency, Huntington	[33, 106]
*Twin of Hotdog*	Enhancer RNA	*HoxD*	Nucleus	Enhancer	Development	[179]
*UCA1*	LincRNA	*OR10H1*	Cytoplasm	Post transcriptional	Senesence	[162]
*Xist*	LincRNA/antisense	*Tsix*	Nucleus	Transcription factor, histone modification, chromatin remodeling	X-chromosome inactivation	[40, 71, 120, 121, 182, 183]

### Molecular and genetic structure of LncRNAs

Like mRNAs, lncRNAs are transcribed by Polymerase II, mostly 5′-capped, polyadenylated and spliced, though on average they contain a lower number of exons than mRNAs and their expression level assessed across different tissues is lower [3, 4]. There are various algorithms calculating the coding probability based on the length of a potential open reading frame (ORF), the similarity of such an ORF to known protein-coding genes, frequency of in-frame nucleotide hexamers or other empirical sequence features [5]. In general, RNA transcripts containing short (<100 nt) non-conserved ORFs, which have no homology to known peptide sequences and do not match to peptides identified in mass spectrometry screens are considered noncoding [6]. Interestingly, the majority of lncRNAs are associated with ribosomes [7, 8], though they do not show the characteristic release of ribosomes [9] or the typical 3-nucleotide phasing corresponding to codons of an ORF [10, 11]. However, in rare instances, functional oligopeptides have been found to be translated from putative lncRNAs [12–14].

Deep-sequencing experiments revealed many examples of genes producing both protein-coding and noncoding transcripts by alternative splicing. However, so far, only very few reports demonstrate a functional role for both the noncoding RNA(s) and the protein(s) encoded by transcripts derived from the same gene [15, 16]. It is tempting to speculate that such dual usage of transcripts is more frequent than anticipated.

RNA molecules have the potential to form highly structured macromolecules by folding into double-stranded stems, single-stranded loops and bulges, which again can fold further into three-dimensional structures, allowing for the potential formation of complex shapes. So far, the structure of only a few RNAs has been experimentally determined using a combination of chemical assays, and by determining the accessibility of base-paired or single-stranded RNA by various RNases [17, 18]. However, these methods still have the limitation that they can only reveal the secondary, but not the tertiary (3D) structure. In addition, computational predictions are only beginning to provide reliable results, but program learning from experimental data might improve the predictions and more closely mirror experimental observations. This process is accelerated by recently developed techniques combined with high-throughput sequencing, such as SHAPE-MaP and icSHAPE [19, 20].

The similarity between mRNA-encoding and lncRNA genes is furthermore reflected by the chromatin signatures at the genomic regions from where they are transcribed. Their transcriptional start site is, in most cases, marked by the H3K4me3 histone modification and the transcribed region by the H3K36me3 mark, no matter if the lncRNA originates from its own promoter or from an enhancer. However, these histone marks are less prominent than observed at mRNA coding genes, whereas H3K4me1, a characteristic mark of enhancers, tends to be more prominent at genomic regions encoding lncRNAs [2, 21–23]. As expected for transcribed regions, lncRNA promoters correspond with DNaseI hypersensitive sites, which indicates accessible chromatin [24, 25].

**Fig. 1:**
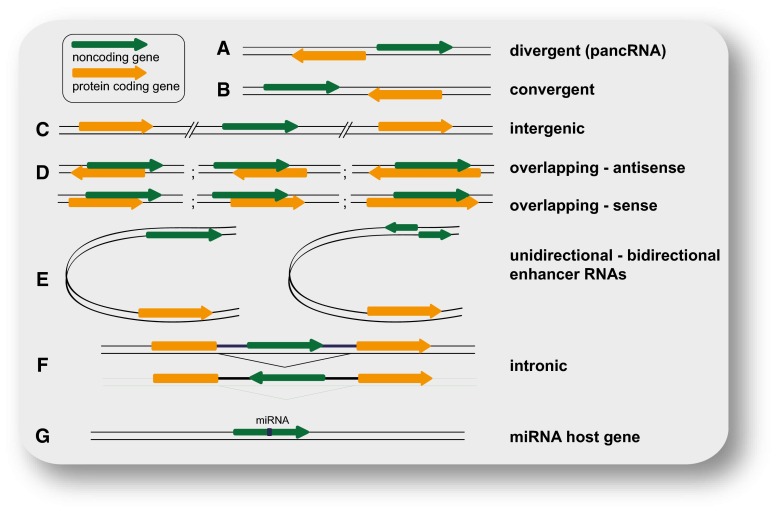
Classification of lncRNAs according to their position relative to neighboring gene(s). **a** Divergently transcribed lncRNA originating from the same promoter region as the adjacent (usually protein coding) gene, but from the opposite strand; **b** convergently transcribed genes encoded on opposite strands and facing each other; **c** intergenic (or intervening) lncRNA (or lincRNA) located distant from other genes (usually >10 kb); **d** examples for various cases of lncRNAs overlapping with other genes on the same or the opposite strand; **e** enhancer RNAs expressed as uni- or bidirectional transcripts; **f** LncRNA transcribed from an intron of another gene; **g** lncRNA hosting a miRNA. Noncoding genes are shown in *green*, protein-coding genes in *orange*

So far, the most commonly used categorization of the highly heterogeneous class of lncRNAs is their position in the genome relative to protein-coding genes (Fig. 1). LncRNAs can be intergenic (lincRNAs) or divergently transcribed from the same promoter as a protein-coding gene (pancRNAs). LincRNAs expressed from a promoter (with high H3K4me3 to H3K4me1 ratio) are classified as promoter-associated lncRNAs (plncRNAs). However, lncRNAs can also be transcribed from enhancers and are then termed eRNAs. They are mostly transcribed in both directions, in contrast to enhancer-associated lncRNAs (elncRNAs), which are unidirectionally transcribed from a promoter with low H3K4me3 to H3K4me1 ratio [23]. Transcription of lncRNAs can also originate from within introns, or overlap with other transcripts in sense or antisense orientation [3]. Some lncRNAs are generated by backsplicing from introns of mRNAs or other lncRNAs and are thus circular (circRNAs) [26, 27].

Genome-wide sequencing of RNA species isolated from cytosolic or nuclear fractions of cells has shown that the majority of lncRNAs tend to be localized in the nucleus or are associated with chromatin, while a considerable fraction localizes to the cytoplasm [3, 28, 29]. The subcellular localization is a good indication of the putative function of a lncRNA, since in contrast to protein-coding mRNAs, lncRNAs can already function while transcription is occurring. In fact, many nuclear and, in particular, chromatin-retained lncRNAs co-regulate transcription and/or chromatin structure at or close to their site of transcription, i.e. in *cis*.

### Conservation and evolutionary aspects of LncRNA

The genomic sequences of lncRNAs are, in general, less conserved than exons, but more than introns of protein-coding genes, pointing towards a rapid evolution with moderate constraints [21, 30]. In contrast to the actual sequence, splice sites of lncRNAs have been found to be more stable during evolution [31, 32]. Despite poor RNA sequence conservation, lncRNAs have frequently been identified across species in syntenic genomic regions [33, 34]. It is therefore quite likely that evolutionary conservation of lncRNAs is embodied by a conserved 3D structure, although this is difficult to assess with current methods. A further layer of conservation is functional conservation between lncRNAs playing equivalent roles in particular biological settings [35].

A different explanation for low conservation of lncRNAs is the increasing number of such transcripts in increasingly complex species. This finding, in combination with the observed highly tissue-specific expression of many lncRNAs, suggests that lncRNAs might be key molecules promoting species-specific features and organ complexity [36–38]. Thus, lncRNAs very likely have played important roles in the evolution of complex organisms.

## LncRNAs regulate gene and genome activity

**Fig. 2:**
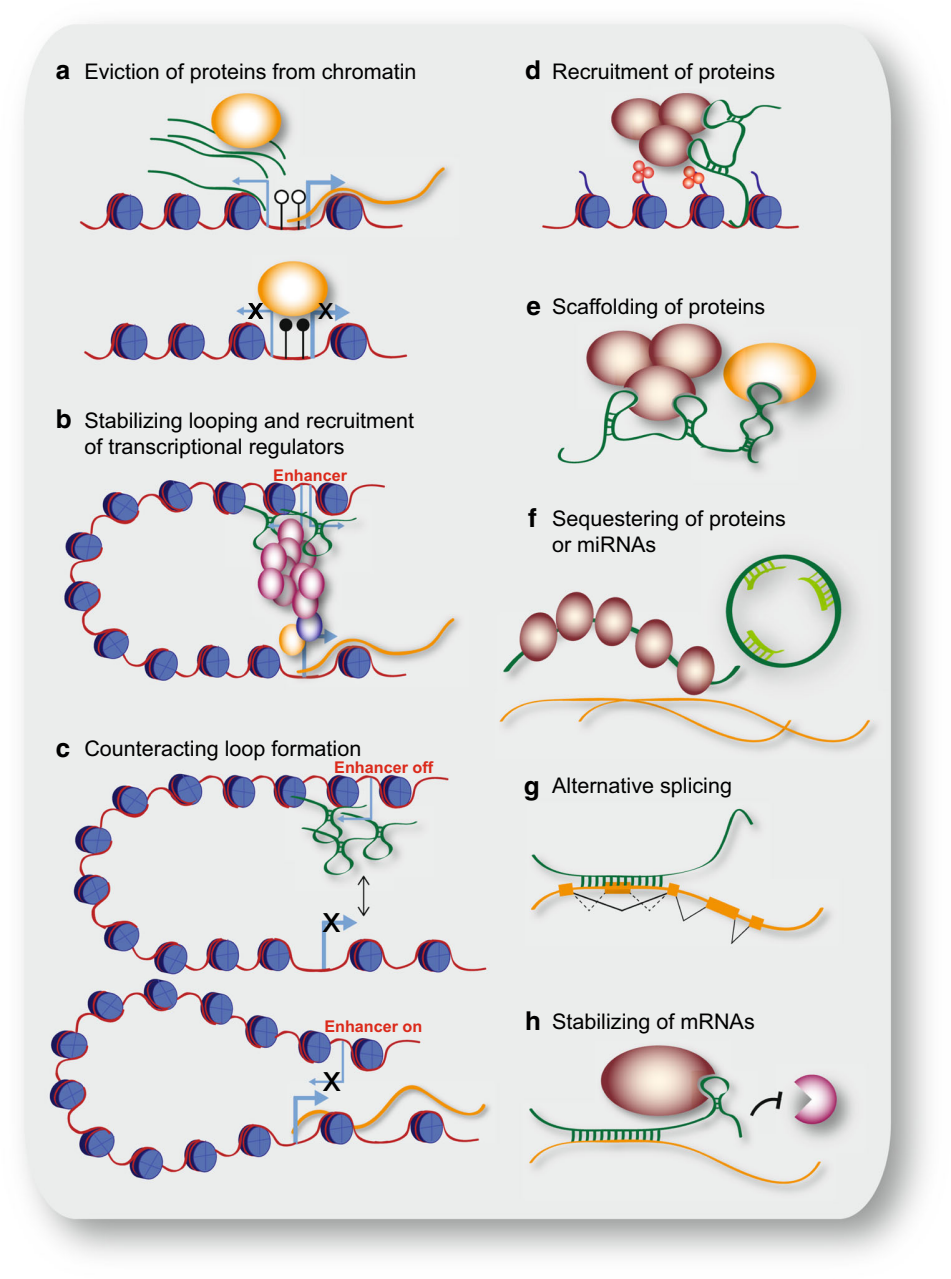
Schematic representation of cellular mechanisms involving lncRNAs. **a** LncRNA transcripts evicting proteins from chromatin; here, pancRNAs prevent DNMT from methylating DNA in their promoter region, thereby ensuring mRNA transcription. **b** LncRNAs recruiting the Mediator complex to an enhancer region, stabilizing loop formation and transcription of the associated gene. **c** LncRNAs transcribed from an enhancer region interfering with enhancer-promoter contact, thereby inhibiting transcription of the protein-coding gene. **d** LncRNA recruiting proteins, such as chromatin-modifying complexes to specific target sites in the genome, e.g. via DNA-RNA triplex formation. **e** LncRNA acting as scaffold linking different proteins required for concerted action. **f** LncRNA binding and sequestering proteins to prevent or attenuate their action, e.g. binding to mRNAs (*left*); circRNA sequestering miRNAs to prevent their binding to mRNAs (*right*). **g** Example of a lncRNA changing the splicing pattern by binding to a primary RNA transcript. **h** LncRNA stabilizing a mRNA by recruiting proteins such as STAU1, thereby preventing degradation

The best-described function for lncRNAs in the nucleus is their role in regulating gene and genome activity on various levels (Fig. 2a–e). Many possible mechanisms by which lncRNAs influence chromatin modifications and chromatin structure, and thereby influence transcription or other chromatin-related functions by epigenetic mechanisms have been investigated. In the following section we will address different levels of gene and genome regulation separately.

### Histone modifications regulated by LncRNAs

Prominent examples of histone-modifying complexes interacting with lncRNAs are the two polycomb repressive complexes, PRC1 and, in particular PRC2, which mediates methylation of lysine 27 on histone 3 (H3K27me), a histone mark associated with repressed or poised genetic loci. The first report of an interaction of PRC2 with a noncoding RNA came from studies on X-chromosome inactivation in mammals, which involves X-inactive specific transcript (*Xist*), a lncRNA that is highly expressed from the inactive X-chromosomes in females (Xi) and recruits PRC2 to the Xi to silence gene expression [39–41].

Another prominent example is *Hotair*, a conserved lncRNA that is transcribed from within the *HoxC* gene cluster. *Hotair* was shown to play a repressive role at the *HoxD* locus by interacting with PRC2. In addition, *Hotair* also interacts with the histone H3K4me1/2 demethylase LSD1 (KDM1), which removes a histone mark of active chromatin and thus reinforces the establishment of a repressive chromatin environment on target loci [42] (Fig. 2d, e). Targeted inactivation of *Hotair* in mice leads to de-repression of imprinted genes and of *HoxD* genes, which lose H3K27-methylation and gain H3K4-methylation marks, as expected from loss of the enzymatic activity of PRC2 and LSD1 at these loci. Furthermore, *Hotair* knockout mice show skeletal homeotic transformation phenotypes, which are typical for mutations affecting Polycomb mediated repression [43]. Interestingly, mice lacking most of the *HoxC* cluster including *Hotair* do not display these phenotypes, suggesting a complex interplay of multiple genomic regions in regulating *HoxC* cluster gene expression [44].

*Braveheart* (*Bvht*) is a lncRNA that is activated during early cardiac differentiation and acts upstream of MESP1, a basic Helix-loop-helix transcription factor involved in early heart development. *Bvht* also exerts its function via an interaction with PRC2 [45]. The lncRNA Fetal-lethal developmental regulatory RNA (*Fendrr*; or *Foxf1* adjacent noncoding developmental regulatory RNA) is likewise involved in cardiac development and heart function and interacts with PRC2. *Fendrr* also interacts with WDR5, which is well known for its presence in the MLL complexes that mediate H3K4 methylation, a mark that is thought to oppose H3K27me. *Fendrr* might play a role at poised genes, or is involved in tuning the balance of active and repressive marks at its target gene promoters, thus adjusting the correct expression level of its targets [46, 47]. These examples are only a small selection and many more lncRNAs seem to function in concert with the PRC2 complex; for more examples, see [48–52].

An intriguing mechanism as to how lncRNAs might recruit their protein interaction partners to specific genomic loci is DNA-RNA triplex formation [53], which has been proposed for several lncRNAs forming a complex with PRC2 [46, 54, 55] (Fig. 2d). In this manner, lncRNAs might direct chromatin or transcriptional modulators to specific genomic sites. This could explain, to some extent, the site-specific action of chromatin-modifying complexes, which do not themselves bind to DNA in a sequence specific manner [38, 56].

An example of a lncRNA interacting with the PRC1 complex is Focally amplified lncRNA on Chromosome 1 (*FAL1*). The interaction of *FAL1* with BMI1, an essential subunit of PRC1, regulates its protein stability [57]. The knockdown of *FAL1* in an ovarian cancer cell line led to gene expression changes, slower cell cycle progression and induction of senescence, similar to the effects of the *BMI1* knockdown. This latter phenotype could be partially rescued by knockdown of the senescence-promoting factor *CDKN1A* (p21). Additional ChIP data suggested that BMI1 binds to the promoter of *CDKN1A* and, together with *FAL1*, suppresses the expression of this target and of many other genes.

Whereas *FAL1* acts in *trans* on PRC1, *ANRIL*, a lncRNA transcribed from the *INK4B*-*ARF*-*INK4A* tumor suppressor locus, acts in *cis*. The *ANRIL* transcript was discovered in a family with inherited melanoma-neural system tumors and assumed to play a role in tumorigenesis [58]. *ANRIL* recruits CBX7 (a PRC1 component) via a POLII-dependent mechanism to its locus in order to repress the neighboring *INK4B*-*ARF*-*INK4A* genes, antagonize cellular senescence and indirectly promote cell cycle activity [58, 59].

The repressive H3K27-methylation, catalyzed by EZH2 in the PRC2 complex, is opposed by MLL complexes, which mediate methylation of H3K4me2/3, a mark associated with loci that are actively transcribed or primed for activation. WDR5 is an integral part of all MLL complexes, but also interacts with several other protein complexes [60]. WDR5 has been found to interact with more than 200 lncRNAs in mouse embryonic stem cells (mESCs) [61]. These interactions are important for WDR5 binding to chromatin since a point mutant that cannot bind RNA also fails to stably associate with chromatin. More specifically, two lncRNAs, *HOTTIP* and *NeST* have been described to recruit WDR5 to their neighboring genes and thus enhance their transcription [62, 63].

### LncRNAs modulate DNA methylation

Whereas many lncRNAs associating with Polycomb complexes act by promoting PRC occupancy at genomic target sites, lncRNAs described in the context of DNA methylation have mostly been found to oppose this epigenetic mark. Frequently, transcription of noncoding RNAs, which interact with DNMTs, keeps a locus free of DNA methylation [54, 64–66] (Fig. 2a). Initially, it was observed that knockdown of extracoding CEBPA (*ecCEBPA*), a transcript starting upstream of the *CEBPA* gene, leads to down-regulation of *CEBPA* and increased DNA methylation of the locus. A subsequent global analysis of all lncRNAs bound to DNMT1 [64] revealed that loci whose RNA products interact with DNMT1 show lower levels of DNA methylation than other loci. A similar observation was made in oocytes and two-cell stage embryos [66], in which the down-regulation of several divergently transcribed promoter-associated ncRNA (pancRNA) led to lower expression of the adjacent protein-coding gene and higher DNA methylation. In the case of one particular gene, *IL17b*, the pancRNA knockdown resulted in cell death of the developing blastocyst. Supplementing the embryos with IL17b protein rescued the phenotype.

In the case of *Tcf21 antisense RNA reducing DNA methylation* (*TARID*), keeping the protein-coding gene (*Tcf21*) on the opposite DNA strand free of DNA methylation required GADD45A and the TET proteins [65] pointing towards a mechanism involving active DNA demethylation.

### LncRNAs regulate chromatin remodeling

In addition to interaction with enzymatic complexes, which covalently modify chromatin, lncRNAs have also been shown to control chromatin remodeling complexes that can alter the nucleosome spacing in an energy dependent manner. In human prostate cancer, gene expression analyses showed that SWI/SNF and the lncRNA *SChLAP1* have opposing roles. *SChlAP1* interacts with the SNF5 subunit of the chromatin remodeling complex SWI/SNF, and globally inhibits binding of SWI/SNF to chromatin, subsequently leading to genome-wide de-repression of gene activity [67]. Similarly, the lncRNA *Myheart* (*Mhrt*), a lncRNA transcribed divergently to *Myh6* and overlapping with *Myh7* in antisense direction, binds to BRG1, the ATPase subunit of the SWI/SNF complex, and excludes it from the *Myh6/7* locus, thus preventing chromatin remodeling [68]. BRG1, in turn, down-regulates *Mhrt.* Cardiac stress leads to upregulation of BRG1 to a level allowing it to overcome the repulsion of the SWI/SNF complex from chromatin by *Mhrt* and to bind to the *Myh6* locus.

The *Evf2* lncRNA, transcribed from the genomic region between *Dlx5* and *Dlx6*, promotes SWI/SNF binding to the enhancers of these genes. However, *Evf2* inhibits the remodeling activity of SWI/SNF and thus interferes with upregulation of *Dlx5/Dlx6*. Accordingly, in *Evf2* mutant mice, *Dlx6* and, to a lesser extent, *Dlx5* are upregulated [69, 70]. In a more recent study, it was observed that the binding of *Evf2* to BRG1 can be out-competed by other RNAs of similar length and results in reduced remodeling activity, suggesting that the binding of lncRNAs to the SWI/SNF complex is promiscuous. *Xist*, on the other hand, binds to components of the SWI/SNF complex, but excludes the complex from the Xi, rather than recruiting it [71]. The SWI/SNF complex can also be recruited by lncRNAs and mediate gene activation, as has been found in hepatocellular carcinoma cancer stem cells. A lncRNA, *lncTCF7* or *WNT signaling pathway activating non*-*coding RNA* (*WSPAR*), transcribed 200 kb upstream of *TCF7*, can recruit SWI/SNF to the *TCF7* promoter and thus activate *TCF7* expression and WNT signaling [72].

### Is the observed binding of numerous lncRNAs to chromatin modifiers specific or promiscuous?

The specificity of lncRNA interactions with chromatin-modifying complexes has been questioned. For instance, a large number of lncRNAs have been shown to co-purify with PRC2, possibly suggesting non-specific binding [50, 52, 73]. Similarly, 15 % of all lncRNAs present in a particular cellular context were shown to be bound by DNMT1 [64, 74]. Likewise, WDR5 binds to more than a thousand RNAs in mESCs, of which 20 % are lncRNAs [61]. This issue of promiscuity was assessed exemplarily for PRC2 in a systematic biochemical study [75], where the authors demonstrate that PRC2 binds RNA in a promiscuous manner, but has remarkably higher affinity for specific lncRNAs than for e.g. bacterial mRNA. Furthermore, they provide evidence that the binding strength correlates with the length of the RNA. Though these studies were performed in vitro at non-physiological conditions, they suggest that the chromatin-modifying complexes can sufficiently differentiate between specific and non-specific interactors. Moreover, since most lncRNA transcripts are expressed at few copies per cell, it is likely that the number of PCR2 molecules per cell is in excess to the number of lncRNA transcripts, which contain a high affinity site for PRC2. Overall, it is not unexpected that a chromatin-modifying complex acting at numerous genomic loci binds many different individual lncRNAs, which stabilize it or recruit it to distinct genomic loci.

### Interactions of lncRNAs with transcription factors

Besides chromatin modifiers, transcription factors have also been found to interact with lncRNAs. The lncRNA *definitiveendoderm*-*associated lncRNA1* (*DEANR1*) (also known as *ALIEN*), for instance, is transcribed downstream of *FOXA2* and is highly induced during differentiation of ESCs towards definitive endoderm [76, 77]. Knockdown of *DEANR1* inhibits endodermal differentiation due to the reduction of *FOXA2* expression. This interpretation is strongly supported by the fact that endoderm differentiation is rescued in *DEANR1* knockdown cells upon forced *FOXA2* expression. Mechanistically, *DEANR1* was proposed to interact with SMAD2/3 and to recruit it to the *FOXA2* promoter.

The lncRNA *rhabdomyosarcoma associated transcript* (*RMST*) is required for neuronal differentiation by mediating binding of the transcription factor SOX2 to approximately half of its binding sites [78]. Certain lncRNAs have been linked in different ways to transcription factors of the NF-κB pathway. *Lethe* (*after the ‘river of forgetfulness’* in Greek mythology) interacts with RelA and prevents it from binding to DNA [79]. *p50*-*associated cyclooxygenase*-*2 (COX*-*2) extragenic RNA* (*PACER*), on the other hand, sequesters p50, which forms homodimers and is repressive at high concentration, thereby lowering its concentration and allowing it to form activating heterodimers with p65 [80]. Another lncRNA, *lnc dendritic cells* (*lnc*-*DC*) binds to the transcription factor STAT3 in the cytoplasm and prevents its dephosphorylation by SHP1, thereby activating STAT3 and thus dendritic cell differentiation [81].

An elaborate mechanism linking transcription factor binding and chromatin modifications has been found for breast cancer anti-estrogen resistance 4 (*BCAR4*). This lncRNA binds to the SMAD nuclear interacting protein 1 (SNIP1) and a phosphatase (PNUTS) involved in RNA polymerase II regulation. In response to cytokine stimulation, *BCAR4* lifts the inhibitory effect of SNIP1 on p300, a histone acetyl transferase and transcriptional activator. Acetylated histones are necessary for *BCAR4* mediated recruitment of PNUTS, which in turn leads to active polymerase II at GLI2 controlled genes [82].

Also CTCF, a protein with the dual function of a transcriptional regulator and a boundary factor, has been shown to interact with numerous noncoding RNAs [83]. An example is the interplay between CTCF and the lncRNAs *Jpx*, *Tsix* and *Xite*, which is important for X-chromosome inactivation [83, 84]. Prior to inactivation, *Tsix* and *Xite* recruit CTCF in *cis* to the X-inactivation center promoting homologous X-chromosome pairing at the Xic [83]. During X-chromosome inactivation, *Jpx* evicts CTCF in *trans* from the *Xist* locus and thereby prevents promoter blockage by CTCF [83]. These examples illustrate the dynamic interaction between lncRNAs and transcription factors in regulating chromatin-mediated cellular functions.

### Regulation of genome organization by looping of enhancers

Previously, transcripts found at enhancers had been interpreted as byproducts of transcription from promoters contacted by enhancers. Now, it is well established that RNA transcripts produced at active enhancers are functionally important [85–87]. An example of enhancer-linked transcripts, already identified two decades ago, has been described for the locus control region (LCR) of the *β*-*globin* cluster [88, 89]. To date, numerous studies have assigned functions to enhancer-associated RNAs [90] (Fig. 2b). There are different classes of RNAs observed at enhancers. The classical enhancer RNAs (eRNAs) are derived from bidirectional transcription, are unspliced and rather short transcripts [85, 91]. Additionally, there are also unidirectional, often spliced and processed transcripts, which are indistinguishable from canonical lncRNAs, but happen to be transcribed from an enhancer [23, 92]. These transcripts often function in *cis*. Finally, there are lncRNAs with enhancer-like functions (also called ncRNA-a) [93]. Whether this classification reflects different functional classes of RNAs remains to be investigated.

In general, eRNAs are co-regulated with their neighboring gene(s) [91] and presumed to be important for gene expression, possibly by supporting cohesin binding [94]. However, experimental support for the latter is rather mild. While eRNAs in some studies were shown to stabilize chromosome loops [94, 95], they appeared to be dispensable for promoter-enhancer contact in others, raising doubts about the role of eRNAs in chromosome looping [96, 97].

LncRNAs transcribed from enhancer regions can also have inhibitory effects on their target gene. The promoter deletion of the lncRNA *Haunt* (also known as *linc*-*Hoxa1*), for instance, has been shown to lead to upregulation of several genes of the neighboring *HoxA* gene cluster. However, larger deletions in the *Haunt* locus impair *HoxA* expression [98], demonstrating that the genomic locus can have opposing functions to the RNA product encoded by it. The repressive effect of *Haunt* was furthermore observed at the single cell level confirming that the expression of *HoxA1* and *Haunt* are anti-correlated [99].

A similar mechanism was found for *Playrr* (*D030025E07Rik*), a lncRNA encoded upstream of the homeodomain transcription factor *Pitx2* [100]. *Pitx2* expression is constrained to the left side of the gut dorsal mesentery, whereas expression of *Playrr* is detected on the right, but spreads to both sides in *Pitx2* deletion mutants. A CRISPR/Cas9 generated mutation resulting in *Playrr* RNA decay caused upregulation of *Pitx2* expression [100]. Both experiments reveal a mutual inhibitory interaction between *Pitx2* and *Playrr*. An enhancer involved in *Pitx2* regulation in the gut overlaps the TSS of *Playrr*. In the cells on the left-hand side, where *Pitx2* is expressed, the *Pitx2* locus was found to be in closer proximity to the *Playrr* locus than in cells on the right side and to cause down-regulation of *Playrr* by an unknown mechanism. In cells on the right, on the other hand, *Playrr* RNA is thought to cause a separation of the *Pitx2* locus from the enhancer at the *Playrr* locus suggesting that *Playrr* expression interferes with the looping of the *Pitx2* promoter to its enhancer at the *Playrr* locus and thus with *Pitx2* gene activation (Fig. 2c).

Furthermore, the lncRNAs *PRNCR1* and *PCGEM1* promote enhancer-promoter looping in prostate cancer cells [101]. Other lncRNAs have been suggested to assist chromosome conformation more globally. A particular situation can be observed at the inactive X-chromosome (Xi). The 3D-structure of Xi is dependent on *Xist*, supposedly because *Xist* excludes cohesin binding from Xi [71]. Deletion of *Xist* led to loss of the compact chromosome structure of the Xi and reorganization into chromosome domains as they are found on the active X-chromosome. Recently, another lncRNA, functional intergenic repeating RNA element (*Firre*), which escapes X-chromosome inactivation was suggested to stabilize the perinucleolar location of the Xi [102]. Additionally, *Firre* was proposed to mediate focal trans-chromosomal contacts [103]. This finding was questioned by a different study, which reported a dispersed nuclear distribution of *Firre* and a role in the control of factors involved in RNA processing [104]. These examples show that lncRNAs can play important roles in controlling local intra- and inter-chromosomal genomic structure and interactions.

### Regulation at the posttranscriptional level

LncRNAs primarily found in the cytosol are thought to be involved in gene regulation on the posttranscriptional level (Fig. 2e–h). Accordingly, several lncRNAs have been shown to influence splicing patterns of either specific genes or globally by interacting with splicing factors. The lncRNA *Pnky* (divergent to *Pou3f1*) binds to the splicing factor PTBP1 and thereby regulates the splicing patterns of a subset of genes involved in neurogenesis [105]. Whether *TUNA* (*megamind*), which interacts with PTBP1 as well, functions via the same mechanism remains to be investigated [33, 106]. In addition, myocardial infarction associated transcript (*MIAT*) has been found to influence splicing in different systems. In embryonic neurogenesis, *MIAT* is involved in controlling the splicing pattern of *Wnt7b* [107]. In iPS cells and mouse primary neurons, *MIAT* binds to splicing factors, and in post-mortem brains of schizophrenia patients, it was found to be down-regulated, suggesting that it might contribute to this pathological phenotype [108]. *MALAT1*, a highly expressed lncRNA enriched in nuclear speckles, has been suggested to be a general component of the splicing machinery [109]. However, this view has been challenged since the *MALAT1* knockout did not show disintegration of splicing speckles [110]. A very specific role in the cytoplasm was found for the lncRNA non-coding RNA activated by DNA damage (*NORAD*) [111]. *NORAD* sequesters PUMILIO proteins and thereby promotes genomic stability (Fig. 2f). Ablation of *NORAD* leads to hyperactivity of PUMILIO causing chromosome instability and aneuploidy due to repression of PUMILIO target mRNAs that function in DNA replication, mitosis and DNA damage repair.

The most recently described class of lncRNAs that came into focus are the circRNAs. Due to their circular structure, they are resistant to degradation by exonucleases and are therefore thought to be highly stable within a cell. The brain specific circRNA *CDR1as* was shown to act as a sponge for the miRNA *let*-*7* [26, 27] (Fig. 2f). Accordingly, overexpression of *CDR1as* in zebrafish caused impaired midbrain development, similarly to the effect of a *let*-*7* inhibitor. It was also shown that small polypeptides can be derived from some circRNAs, extending the functional repertoire of these stable RNA molecules [112]. Most likely, circRNAs do not only interact with other transcripts, but also with proteins and thus may function in a wide range of different processes.

## Functions of lncRNAs in development and differentiation

In the following section, we will focus on the physiological effects of lncRNAs in the organism, during embryonic development and in in vitro differentiation systems. There are still few known examples of lncRNAs shown to play important roles in vivo, and thus we will revisit some of the lncRNAs already mentioned above.

Maternal transcripts present in oocytes and zygotes contribute to early differentiation processes. In a single-cell sequencing study examining oocytes, zygotes and cells from the first cell divisions of the early mouse embryo, lncRNA transcripts were found at all stages examined [113]. One subclass of lncRNAs, the pancRNAs, was compared between murine two-cell embryos and oocytes [66]. A subset of pancRNAs found to be expressed in two-cell embryos but not in oocytes was found to be important for counteracting DNA methylation at the adjacent promoters thereby ensuring expression of the neighboring gene.

The mechanisms first found to be linked to lncRNAs were imprinting and X-chromosome inactivation. Accordingly, the first lncRNA that was genetically targeted and deleted was *H19*, an imprinted gene that was already suspected to be a lncRNA 25 years ago [114]. *H19* is exclusively expressed from the maternal allele and is inhibitory for the adjacent gene, Insulin-like growth factor 2 (*Igf2*) [114–116]. An *H19* deletion on the maternally inherited chromosome led to an increase in *Igf2* expression and thus to increased bodyweight. This phenotype could be rescued by deletion of one *Igf2* allele. *H19* knockout mice are viable and fertile [116], but have reduced muscle regeneration capacities due to loss of two miRNAs encoded within the *H19* transcript [117]. The imprinted *Igf2r* gene is silenced by the lncRNA *Airn*, which is transcribed from the opposite strand, overlapping the 5-prime region of *Igf2r*. Here, silencing is not achieved by the lncRNA transcripts as such, but by transcription through the *Igf2r* promoter, which interferes with RNA PolII recruitment [118]. Several further lncRNAs with a well-established role in imprinting have been reported. For details we would like to refer to a recent review [119].

Insight into the developmental function of *Xist* was obtained two decades ago when *Xist* knockout mice were generated and analyzed [120, 121]. Male mice lacking *Xist* are unaffected, whereas females die during the first half of gestation. Interestingly, knockout females with a single X-chromosome (XO) lacking *Xist* are healthy, strongly suggesting that it is not the lack of *Xist*, but the failure to adjust the X-chromosome gene dosage that causes embryonic lethality.

In a more recent example, the deletion of another imprinted lncRNA gene in the mouse, *Gtl2* (*Meg3*, *maternally expressed 3*), showed clear *cis*-acting effects on neighboring genes resulting in perinatal lethality [122].

Many other mouse models in which lncRNAs have been removed by deletion of genomic fragments did not show overt phenotypes, but after detailed analyses, some specific defects have been observed [110, 123–126]. For instance, deletion of *NEAT1* resulted in viable and fertile mice, however, knockout females showed about 50 % reduced fertility due to reduced progesterone production and corpus luteum formation [127]. Ovary transplantation or progesterone administration rescued offspring rates, confirming the cause of the phenotype, though the molecular mechanism remains to be determined.

*Fendrr* is one of few rare examples of lncRNAs demonstrated to play an essential role in organ development and embryo survival. *Fendrr*, which is divergently transcribed from the *Foxf1* promoter, was analyzed by two different strategies [47, 123]. In one study, the insertion of a transcriptional stop cassette replacing the first exon of *Fendrr* leaving most of the genomic context undisturbed resulted in impaired heart function, omphalocele (defects in body wall development), and embryonic death [47]. *Fendrr* interacts with chromatin-modifying complexes and with DNA, possibly through triplex formation, and thereby changes the chromatin landscape of specific target promoters in *trans*, in particular those of mesodermal transcription factors [46]. A BAC-transgene expressing a single dose of *Fendrr* rescued the developmental defects caused by the loss of *Fendrr*, strongly suggesting that the observed phenotype is caused by lack of *Fendrr* RNA, and not by disruption of genomic sites or promoter elements of the adjacent *Foxf1* gene. This conclusion was supported by a different knockout strategy, in which the first exon was left intact and which resulted in perinatal death [123]. In the early embryo, *Fendrr* is only transiently expressed in nascent lateral mesoderm [47]. However, later during development it is expressed in the lungs, a derivate of endoderm and lateral mesoderm, where it again might play an essential role [123, 128].

More lncRNAs involved in mesoderm and endoderm development have been reported. For instance *DEANR1* (also known as *ALIEN*) is expressed downstream of the endoderm master regulator *FOXA2* [76, 77]. *DEANR1* depletion results in reduced expression of *FOXA2* and accordingly to reduced differentiation of hESCs towards definitive endoderm. Interestingly, this defect can be rescued by restoring normal levels of *FOXA2* expression, suggesting that *DEANR1* functions mainly in *cis*. Another study described the same lncRNA, here called *ALIEN*, in cardiovascular progenitor cells in mouse and zebrafish. Depletion of *ALIEN* interfered with cardiac development, although the functional mechanism remains to be investigated further. *DEANR1* was described to be localized in the nucleus, whereas *ALIEN* was seen in the perinuclear space and in the cytoplasm, even though both were analyzed in differentiating human stem cells. The discrepancy between the two studies might be a consequence of modifications in the differentiation method [76, 77].

Also, the transcription factor *Pitx2*, which is essential for the development of many different organs, especially during the establishment of left–right asymmetry, is regulated by a lncRNA in the embryonic gut [100]. In this case, the lncRNA *Playrr* is transcribed from a distant known enhancer region. The expression patterns of *Pitx2* and *Playrr* are negatively correlated due to interference of *Playrr* with *Pitx2* activation (for details see above).

LncRNAs expressed during neuronal and brain development have attracted particular attention. As described earlier, many lncRNAs are highly specifically expressed in distinct cell types, as illustratively shown in a targeted LacZ reporter screen [37] and in expression screens [36, 107, 129, 130]. A number of lncRNAs located close to the *PouIII* genes *Pou3f3* and *Pou3f2* (*Brn1* and *Brn2*) encoding important neuronal transcription factors, were also found to play roles in neuronal development [37, 105, 123, 131]. The lncRNA *Dali* (or *Dalir*) (DNMT1-Associated Long Intergenic RNA) is transcribed 50 kb downstream of *Pou3f3.* The depletion of *Dali* leads to a reduction of *Pou3f3* expression and to impaired neuronal differentiation [131]. Gene expression analysis upon knockdown and genome-wide localization (CHART) revealed POU3F3 dependent and independent targets of *Dali*. Furthermore, *Dali* physically interacts not only with various chromatin modifiers, but also with POU3F3 itself suggesting a multilayered association between *Dali* and POU3F3. The replacement of *Pantr1* (*POU domain*, *class 3*, *transcription factor 3 adjacent noncoding transcript*), which is divergently transcribed to *Pou3f3*, by insertion of the lacZ gene did not influence *Pou3f3* transcription itself, but modulated other *Pou3f* transcription factors in *trans* [37]. *Pantr2* (or *Linc*-*Brn1b*), expressed convergent with *Pou3f3*, did not have this effect on *Pou3f* factors, and upon deletion led to fewer intermediate progenitors in the developing cortex. Thus, each of these three different lncRNAs, which are all transcribed near the *Pou3f3* locus, affect neuronal development yet through distinct mechanisms.

Furthermore, another lncRNA located in a region important for neural development is *Evf2* in the *Dlx5/6* locus [69]. *Evf2* was targeted by inserting a polyadenylation signal close to the promoter without deleting genomic DNA. *Evf2*^−/−^ embryos have less GABAergic neurons than wild type controls. This mutant effect could be rescued by in utero electroporation of *Evf2* RNA, confirming that the phenotype is indeed dependent on this lncRNA.

*RMST*, also termed *non-coding rhabdomyosarcoma* (*NCRMS*) in humans, is a noncoding transcript expressed in rhabdomyosarcomas [132] and in developing dopaminergic neurons during murine development [133]. *RMST* is induced during neuronal differentiation of hESCs, and its depletion interferes with neuronal differentiation via its binding partner SOX2 (see above) [78, 107, 129]. Interestingly, *RMST* was found associated with obesity in a GWAS study, possibly pointing to a role of *RMST* in a neuronal regulatory network influencing energy balance [134].

Two more lncRNAs involved in nervous system development are the *retinal noncoding RNA2* and *4 (RNCR2* and *RNCR4).* They were first found to be expressed in the developing retina and later also assigned a functional role in this process [135]. *RNCR4* is part of a network including the RNA helicase DDX3X and the *miR*-*183/96/182* that is essential for retinal architecture [136]. *RNCR2*, also known as *MIAT* or *Gomafu*, interferes with retinal cell differentiation when overexpressed or depleted [135, 137]. Furthermore *MIAT* is expressed earlier in the developing brain, where it localizes to the nuclear matrix [138]. Purifying distinct subpopulations of the developing brain at E14.5 revealed that *MIAT* is highly abundant in differentiating progenitors, where it is involved in corticogenesis by regulating the splicing pattern of *Wnt7b* [107] (Fig. 2g).

Another ectodermal derivative, the developing epidermis, has also been shown to be regulated by lncRNAs. During epidermal differentiation and maintenance, the nuclear lncRNA *ANCR* (anti-differentiation ncRNA) is essential for maintaining the proliferating progenitor pool by repressing differentiation factors, amongst them the transcription factors MAF and MAFB, probably in a PRC2 linked manner [139–141]. Depletion of *ANCR* leads to differentiation and loss of progenitor cells. Its differentiation promoting counterpart is a cytosolic lncRNA, terminal differentiation induced ncNRA (*TINCR*) that interacts with STAU1 and as a protein-RNA complex stabilizes mRNAs of pro-differentiation genes including *MAF* and *MAFB* [141, 142] (Fig. 2h). Ectopic expression of MAF:MAFB in *TINCR* depleted cells restored the expression of the differentiation gene signature showing that *TINCR* is crucial for ensuring appropriate protein levels in particular of MAF and MAFB.

## Functions of lncRNAs in pathological settings

LncRNAs as a “new” class of molecules have also attracted attention in pathological settings. Here, we will highlight two very prominent areas, cancer and cardiovascular disease.

### Cancer

Approximately one third of publications currently listed in PubMed linked with the keyword “lncRNA” also contain the keyword “cancer”. Even before the era of next generation sequencing, a few studies found lncRNAs to be dysregulated in tumors [35, 143, 144]. To date, numerous primary tumor samples, metastatic biopsies, the adjacent normal tissue and cancer cell lines have been profiled for their RNA expression and the results recapitulate the high degree of specificity found for noncoding RNAs in normal tissue compared to protein-coding genes [25, 67, 145–147]. Clustering of lncRNA expression patterns identifies subclasses of tumor types with high significance, opening the possibility to define tumor subtypes with a high chance to respond to targeted therapy. A study that considered samples from more than 5000 tumors from the cancer genome atlas (TCGA) project, demonstrated that, while approximately the same number of protein coding and lncRNA genes were dysregulated, 60 % of lncRNAs showed specificity for only one tumor type [146].

In addition, somatic copy number alterations (SCNA) found in cancer tissue often include lncRNA loci [57, 146, 147]. For example, the lncRNA *FAL1*, a lncRNA that was found amplified in a number of different tumor types, was shown to be important for the tumorigenic effects of different cell lines via a BMI1-mediated mechanism (see above), and its expression level and copy number significantly correlated with patient survival [57]. Furthermore, several lncRNAs have been shown to be prognostic for overall or disease-free survival of patients [67, 82, 148–150], information that is integrated into therapeutic decisions. Depletion of selected lncRNAs led to reduced malignancy, assayed as tumor size or metastatic potential in xenograft experiments [67, 72, 82, 101, 148, 149, 151–154], although these experiments were not able to distinguish between effects on tumor formation versus tumor progression. Further refinement was achieved when RNA knockdown was induced after tumor formation, mimicking therapeutic conditions. Reduced tumor growth was, for instance, observed upon *FAL1* depletion [57]. In a different study, the inhibition of *MALAT1* after tumor induction reduced metastasis formation in a xenograft lung cancer model and in a metastasizing breast cancer model [149, 155]. *MALAT1* knockout mice do not show overt phenotypes [110, 156, 157] suggesting that down-regulation of *MALAT1* in humans might also not be harmful for normal human cells, which makes it an attractive target for systemic application in patients.

A special case is the noncoding pseudo gene *BRAFP1*, which has been shown to be either mutated or aberrantly expressed in cancers, like its coding homologue *BRAF*. A mouse model revealed that induced overexpression of *Braf*-*rs1* (the murine homologue of *BRAFP1*) leads to DLBCL (diffuse large B cell lymphomas)-like tumors. However, when the transgene was first induced to form tumors and then shut down again, the tumors regressed demonstrating the functional significance of the pseudo gene [158]. *Braf*-*rs1* was shown to cause upregulation of *Braf* by acting as a ceRNA (competing endogenous RNA) and by “sponging” miRNAs that would otherwise target *Braf* transcripts.

One mechanism that cells can employ to prevent tumor formation is oncogene-induced senescence (OIS) [159]. The *INK4B*-*ARF*-*INK4A* locus, which plays a major role in OIS, is silenced in proliferating cells by Polycomb group proteins. This mechanism is dependent on the lncRNA *ANRIL*, which is transcribed from within the locus and acts in *cis* [58, 59, 160]. Furthermore, the *MIR31HG* gene located 400 kb upstream of the *INK4B*-*ARF*-*INK4A* locus, also encodes a lncRNA involved in the regulation of *INK4A* [161]. A third lncRNA functioning in OIS, *UCA1*, stabilizes mRNAs of protein-coding genes involved in the senescence pathway, probably by sequestering hnRNPKA1 [162]. Also, the tumor suppressor p53 and its associated signaling pathways include lncRNAs. p53 binds and regulates numerous lncRNAs, such as the *linc*-*p21*, which in turn regulates gene expression in concert with p53 and p21 [163–166].

### Cardiovascular diseases

LncRNAs have also been linked to cardiovascular diseases (CVD), which are a major cause of death in western societies, and have been studied both in patient data, as well as in mouse models. It was observed that several genomic regions significantly associated with myocardial infarction encode lncRNAs, for example *MIAT* and *ANRIL* [167, 168]. Deletion of a 70 kb region including a large proportion of the *ANRIL* locus led to increased mortality in mice, particularly in combination with a high fat diet [169].

In other studies, massive parallel RNA sequencing revealed numerous lncRNAs displaying altered expression following surgically-induced myocardial infarction in mice [170, 171]. In cellular differentiation models, the depletion of selected lncRNAs impaired cardiomyocyte formation making them promising candidates for in vivo assessment of their functions [172]. Analysis of the noncoding transcripts originating from the *myosin heavy chain 7 (Myh7*) locus, an important structural protein required for heart contractions, revealed that one, the lncRNA *Mhrt*, was down-regulated after induced myocardial infarction. Ectopic expression of *Mhrt* in mice, which had been subjected to this operation reduced the severity of symptoms observed in control mice during recovery [68]. When lncRNAs in plasma from myocardial infarction patients were profiled, a mitochondrial lncRNA named long intergenic noncoding RNA predicting cardiac remodeling (*LIPCAR*) was found to significantly correlate with mortality caused by CVD and could thus be considered as a biomarker [173]. Whether this lncRNA also plays a causative role in infarction or heart failure remains to be investigated.

LncRNAs have been widely suggested as biomarkers and therapeutic targets in certain pathological settings. One reason for this is the extremely specific expression of many lncRNAs, opening the potential of targeting cellular subgroups rather than affecting patients systemically as in conventional treatments. Even if a lncRNA itself is not the disease driver, it can serve as drug target in cases where it acts as co-factor for a driver gene, which is more broadly expressed. Also, since lncRNAs often play quantitative roles rather than acting as switches in genetic programs, side effects of therapeutic treatments might be easier to control.

## Concluding remarks

In recent years, the analysis of lncRNAs has highlighted the large diversity of cellular functions involving RNA molecules and has greatly expanded the catalog of functions previously attributed to noncoding RNAs. By now, we can conclude that noncoding RNAs play roles at all levels of gene control, including epigenetic mechanisms and nuclear organization, as well as in RNA processing, stability and translation. The recent discoveries of lncRNA functions provide good reasons for the notion that further investigation of lncRNAs bears high chances for the discovery of novel gene regulatory mechanisms in the future.

So far, only few lncRNAs playing an essential role in embryonic development, organogenesis or tissue homeostasis have been reported. Studies based on in vitro differentiation systems can provide important insights into the molecular modes of lncRNA function in local gene control or in regulatory networks. However, with regard to the roles of lncRNAs, for example in lineage decisions and cellular differentiation many findings are hampered by the lack of definitive proof in vivo. In particular, data derived from in vitro differentiated stem cells, whose genomic integrity and quality is hard to control, have to be interpreted cautiously. Also, the type of genetic change introduced into a lncRNA gene/locus for functional analysis (transcription stop, knockin, deletion etc.) needs to be considered carefully in order to draw the right conclusions [174–176]. Since lncRNAs do not only act as transcripts in *cis* or *trans*, but can also be side products of transcription affecting the expression of overlapping genes or accommodate regulatory DNA elements in their genomic loci, the analysis of their exact mode of action and functional roles in development and disease processes is more complex and difficult than that of protein-coding genes. It is now up to the responsibility of journal editors and reviewers to enforce the high standards of investigation this new and exciting field of research deserves.
